# Functional genetic variants of *CTNNBIP1* predict platinum treatment response of Chinese epithelial ovarian cancer patients

**DOI:** 10.7150/jca.48218

**Published:** 2020-09-30

**Authors:** Haoran Li, Lihua Chen, Xiaoxia Tong, Hongji Dai, Tingyan Shi, Xi Cheng, Menghong Sun, Kexin Chen, Qingyi Wei, Mengyun Wang

**Affiliations:** 1 Cancer Institute, Fudan University Shanghai Cancer Center, Shanghai, China.; 2 Department of Oncology, Shanghai Medical College, Fudan University, Shanghai, China.; 3 Department of Epidemiology and Biostatistics, Key Laboratory of Cancer Prevention and Therapy, Tianjin, China.; 4 Key Laboratory of Breast Cancer Prevention and Therapy, Ministry of Education, National Clinical Research Center for Cancer, Tianjin Medical University Cancer Institute and Hospital, Tianjin, China.; 5 Ovarian Cancer Program, Division of Gynecologic Oncology, Department of Gynecology and Obstetrics, Fudan University Zhongshan Hospital, Shanghai, China.; 6 Department of Gynecological Oncology, Fudan University Shanghai Cancer Center, Shanghai, China.; 7 Department of Pathology, Tissue Bank, Fudan University Shanghai Cancer Center, Shanghai, China.; 8 Duke Cancer Institute, Duke University Medical Center, Durham, NC, USA.; 9 Department of Population Health Sciences, Duke University School of Medicine, Durham, NC, USA.

**Keywords:** genetic variants, single nucleotide polymorphisms, ovarian cancer, *CTNNBIP1*, platinum treatment response

## Abstract

Chemotherapy resistance remains a blockade for successful treatment and longer overall survival of patients with epithelial ovarian cancer (EOC). CTNNBIP1 is an inhibitor of β-catenin that is a chemotherapeutic target for EOC treatment. In the present study, we investigated associations between single nucleotide polymorphisms (SNPs) of *CTNNBIP1* and platinum treatment response of Han Chinese EOC patients and subsequently performed functional prediction and validation of the resultant SNPs. We found that* CTNNBIP1* rs935072 AT/TT variant genotypes were associated with platinum treatment response in the multivariate logistic regression analysis of EOC patients. Specifically, the *CTNNBIP1* rs935072 AT/TT genotypes were associated with a decreased risk of developing chemoresistance ([adjusted odds ratio (OR)] = 0.89, 95% confidence interval (CI) = 0.82-0.97 and* P=*0.010), compared with the AA genotype. Further experiments showed that the underlying mechanism for the *CTNNBIP1* rs935072 A>T change in chemotherapy treatment response resulted from a lower binding affinity of miR-27a-3p, thereby leading to up-regulation of the* CTNNBIP1* expression. We further found that overexpression of *CTNNBIP1* sensitized ovarian cancer cells to platinum treatment. Thus, the present study provides evidence that functional variants of *CTNNBIP1* may regulate the expression of *CTNNBIP1,* a possible mechanism affecting platinum treatment response of EOC patients.

## Introduction

Chemotherapeutic drug resistance in tumor cells is presented as a major cause of significant mortality in human malignancies 1, 2, especially in ovarian cancer 3. Currently, the first-line chemotherapy treatment for ovarian cancer is the paclitaxel-platinum combination, which is given every three weeks for six or eight cycles. The platinum-based regimen yields an objective response rate of > 80% (in which 40-60% of the patients had a complete response rate) in the advanced ovarian cancer, but approximately 80% of the patients with an advanced stage had a progression within three years 4 with a median progression-free survival of only about 18 months, which is mainly attributed to the development of chemoresistance-related recurrence 3. Furthermore, most women diagnosed with epithelial ovarian cancer (EOC) develop an acquired chemoresistance in spite of an initial response to the treatment. As a result, chemotherapy resistance remains a blockade for successful treatment and a longer overall survival (OS) in EOC patients 5. Therefore, to have a better management of EOC, it is essential to elucidate the mechanisms of chemoresistance and to identify potential biomarkers to predict chemotherapy response among EOC patients.

It is known that genetic variants are involved in tumor development and patients' survival from drug treatment 6 and thereby considered to participate in the resistance to the anti-cancer drugs 7, 8. For example, genetic variation in biological signaling pathways, including drug uptake and efflux mechanisms, cell cycle, DNA damage repair, apoptosis and glucose metabolism pathways, has been shown to be involved in different treatment responses to chemotherapy in several cancer types 9-11, including EOC 12, 13. However, the underlying genetic mechanisms responsible for such chemotherapy resistance in EOC patients are still poorly understood.

Susceptibility alleles identified from genome-wide association studies (GWASs) easily omit the truly biologically significant ones because of the strict *p*-value standard, so the candidate gene association studies have also been used as a supplementary method to find the missed alleles. Recently, the Wnt/β-catenin pathway was reported to be involved in the chemoresistance of EOC patients, which may be a potential target for chemosensitization 14. Catenin beta interacting protein 1 (CTNNBIP1), also known as ICAT or an inhibitor of β-catenin, prevents β-catenin from forming a complex with the T-cell factor/lymphoid enhancer factor (TCF/LEF) and thus inactivates the transcription of Wnt target genes, thereby negatively regulating the Wnt/β-catenin pathway 15. Furthermore, several studies have suggested *CTNNBIP1* as a tumor suppressor in cancers of the colorectum 16, breasts 17, cervix 18 and stomach 19. Therefore, these findings provide some clues that *CTNNBIP1* may be a chemotherapeutic target in these cancers.

However, few studies explored the role of *CTNNBIP1* in EOC treatment, and, in particular, single nucleotide polymorphisms (SNPs) of *CTNNBIP1* have rarely been investigated for their roles in the response to platinum treatment of EOC patients. Therefore, we hypothesize that functional genetic variants in *CTNNBIP1* are associated with the response to platinum treatment of EOC patients. In the present study, we first used available genotyping data of *CTNNBIP1* SNPs for association analysis and then performed validation of the significant SNPs identified in the multivariate logistic regression analysis, followed by functional analysis of these SNPs.

## Materials and Methods

### Study population

All the patients included in the present study were unrelated ethnic Han Chinese women diagnosed with histologically confirmed EOC, and their DNA samples were obtained from the blood samples collected by the tissue bank of Fudan University Shanghai Cancer Center (FUSCC). The present study included a total of 495 EOC patients consecutively enrolled for Shanghai Ovarian Cancer Study (SOCS) at FUSCC with complete DNA samples between March 2009 and August 2012. Because the present study mainly focused on associations between SNPs and the response to platinum treatment of EOC patients, we excluded those patients who had not undergone any platinum-based chemotherapies. Therefore, 427 patients were left in the final dataset, whose participation in the present study was approved by the Ethics Committee at FUSCC with a written-informed consent obtained from all recruited individuals. This investigation was conducted according to the principles in the Declaration of Helsinki consent.

### Data collection

Clinical characteristics, including age at diagnosis, the International Federation of Gynecology and Obstetrics (FIGO) stage, histology, grade, residue (optimal debulking <1 cm), ascites and platinum treatment response were collected. Primary patients with an early stage (FIGO stage I and II) received a complete staging surgery, while patients with a late stage (FIGO stage III and IV) underwent a cytoreductive surgery. After a primary surgery, all the patients received platinum-based chemotherapy. Those patients who received chemotherapy were divided into a chemosensitive group (relapsed > 6 months after chemotherapy) and a chemoresistant group (relapsed ≤ 6 months after chemotherapy). All the patients were followed up every three months for the first two years, every 6 months for the next three years, and annually for the following years thereafter.

### Genotyping data and quality control

The genotyped data were obtained from a previous GWAS study that used the Illumina HumanOmni Zhonghua-8 BeadChip for genotyping as described previously 20, and we only selected *CTNNBIP1* as the candidate gene for the analysis. Systematic quality control was performed and the exclusion criterion were as follows: 1) a call rate less than 95%; 2) mapping to X or Y chromosomes; 3) a minor allele frequency <0.05; and 4) with Hardy-Weinberg equilibrium *P* <1×10^-5^. In addition, we analyzed the principle component and found no significant principle component to be related with the response to platinum treatment of EOC patients.

### Imputation

Additional SNPs of CTNNBIP1 not on the GWAS chips were imputed using IMPUTE 2.0 (https://mathgen.stats.ox.ac.uk/impute/impute_v2.html). The reference data used for imputation was from the 1000 Genomes Project (phase 3). The imputed genotypes were qualified by excluding SNPs with the following criteria: a posterior probability < 0.9, minor allele frequency <5%, missing genotypes >10% or significant deviations from Hardy-Weinberg equilibrium.

### False-positive report probability and Bayesian false-discovery probability

False Discovery rate (FDR), false-positive report probability (FPRP), and Bayesian false-discovery probability (BFDP) were used to assess the noteworthiness of an observed association between genetic variants and platinum response. The detailed calculation method of FDR 21, BFDP 22 and FPRP 23 were described previously. The stringent FDR method was not optimal for the SNPs under investigation in the present study, because they are in high linkage disequilibrium (LD) as a result of imputation, a threshold of FPRP value less than 0.2 and BFDP value less than 0.8 were considered statistically noteworthy.

### Association analysis

To screen of relevant SNPs associated with platinum response, we used Package GenABEL 24 in R language to perform logistic regression analyses, with correction for co-variables including age, stage, histology, grade, residue, ascites and neoadjuvant chemotherapy. The LD analysis among the obtained SNPs were performed by using Haploview 25. Receiver operating characteristic (ROC) curve was used to estimate the predictive value of genetic variants in combination with clinical variables in additive models. To illustrate the fitness of the model, an area under the curve (AUC) of ROC curves was also calculated.

### Functional annotation of selected SNPs

The online tools RegulomeDB (http://www.regulomedb.org/), SNPInfo (https://snpinfo.niehs.nih.gov/) and Ensemble (http://www.ensembl.org/) were applied to predict putative function of the identified SNPs. Furthermore, the corresponding mRNA expression levels of the identified loci was assessed by using the online tool GTEx database (http://www. gtexportal. org/ home/) 26 as well as data from the 1000 Genomes Project and from the Hapmap3 (phase III, release I) Project. We also used other online tools including MirSNP (http://bioinfo.bjmu.edu.cn/mirsnp/search/), TargetScan (http://www.targetscan.org/vert_71/) and SNPInfo (https://snpinfo.niehs.nih.gov/) to predict potential miRNA binding with the 3'UTR region of the identified loci. The online tools GEPIA (http://gepia.cancer-pku.cn) and Oncomine Database (https://www.oncomine.org/) were also used to explore gene expression levels in different tissues.

### Cell lines and culture

Two established human ovarian cancer cell lines (i.e., IGROV1 and OVCAR-8) were obtained from the Cell Bank of the Eastern China in December 2016. The identities of cell lines were confirmed by DNA profiling (short tandem repeat, STR). All cell lines were used within 6 months after receipt or resuscitation. All cells were cultured in Dulbecco's modified Eagle's medium (DMEM, HyClone, Thermo Scientific, USA) supplemented with 10% fetal bovine serum (Gibco, Life technologies, USA), 100 U/ml penicillin (Biowest, Nuaillé, France), and 100 U/ml streptoc-mycin (Biowest, Nuaillé, France), and were incubated at 37 °C in a humidified atmosphere with 5% CO_2_.

### Cell transfection

To selectively overexpress CTNNBIP1, the recombinant plasmid pENTER-*CTNNBIP1* containing human full cDNA sequence of *CTNNBIP1* was purchased from Vigene Biosciences (Jinan, China). Both ovarian cancer cell lines IGROV1 and OVCAR-8 were infected with the recombinant plasmids. Control cell lines were generated by infection with plasmids containing the empty vector with the same experimental protocol. To generate miR-27a-3p overexpressing cells, miR-27a-3p mimic or their negative control (Biotend, Shanghai, China) was transfected into cells using Lipofectamine 3000 (Life Technologies, Carlsbad, CA) according to the manufacturer's instructions.

### Luciferase reporter assay

The Psi-CHECK2 vector carrying the 3'UTR of *CTNNBIP1* with either rs935072 A or rs935072 T was constructed. IGROV1 and OVCAR-8 cells were transfected with an appropriate dose of constructed test plasmids and renilla luciferase control plasmid in 96-well plates. Forty-eight hours later, luciferase activities were measured by using the Dual Luciferase Assay Kit (Promega, Madison, WI, USA), and renilla luciferase activities were used to normalize the reporter luciferase activities, which were then rescaled to vector control signals equal to unit 1.

### Cell viability assay

To evaluate cell survival fraction under different cisplatin concentrations, we plated 8×10^3^ cells per well in 96-well plates with 100-μl maintenance medium. On the next day, the cells were treated with various concentrations of cisplatin. Cell Counting Kit-8 (Dojindo Laboratories, Kumamoto, Japan) was used to monitor cell viability 48 hours later, and the number of viable cells was assessed by measurement of absorbance at 450 nm by a Microplate Reader (BioTek Instruments, Winooski, VT, USA).

### Colony formation assay

Colony formation Assay was performed as previously described 27.

### Cell apoptosis analysis

Cell apoptosis analysis was performed as previously described 27.

### Reverse transcription quantitative real-time polymerase chain reaction

Total RNAs were isolated from both IGROV1 and OVCAR-8 cells by using the Trizol reagent (Invitrogen, Life technologies, USA) and reversely transcribed into cDNA using the PrimeScript TM RT reagent Kit (Takara Biotechnology, Shiga, Japan). The primer pairs of *GAPDH* were 5'-GGCCTC CAAGGAGTAAGACC-3' (forward primer) and 5'-CAAGGGGTCTACATGGCAAC-3' (reverse primer). The primer pairs of *CTNNBIP1* were 5'-GGGCGGCACCTTCCT-3' (forward primer) and 5'-CTCTGGGGACTCCTGCTTCT-3' (reverse primer). Three independent experiments were performed for final analyses by using the 2^-ΔΔCT^ relative quantification method.

### Western blotting assay

Antibodies against CTNNBIP1 were purchased from Proteintech (Wuhan, Hubei). All the primary antibodies were used at 1:1000 dilutions and secondary antibodies at 1:5000 dilutions. The assay was performed three times as previously described 27.

### Statistical analysis

Genotyping data were extracted by PLink (version 1.09) (http://pngu.mgh.harvard.edu/purcell/plink/) 28. Other statistical analyses were achieved by R language (version 3.2.4). *χ*^2^ test and logistic regression models were used to estimate the main effects of the SNPs on platinum treatment response. The heterogeneity between two groups was assessed by the Cochran's Q-test. The figures were made using GraphPad Prism and phototshop expressed as mean and standard deviation (SD). Statistical analysis was conducted by Student's *t*-test. All the reported *P* values were two-sided, and* P*<0.05 was considered statistically significant.

## Results

### Logistic regression analysis of associations between SNPs and platinum treatment response

**Figure 1:**
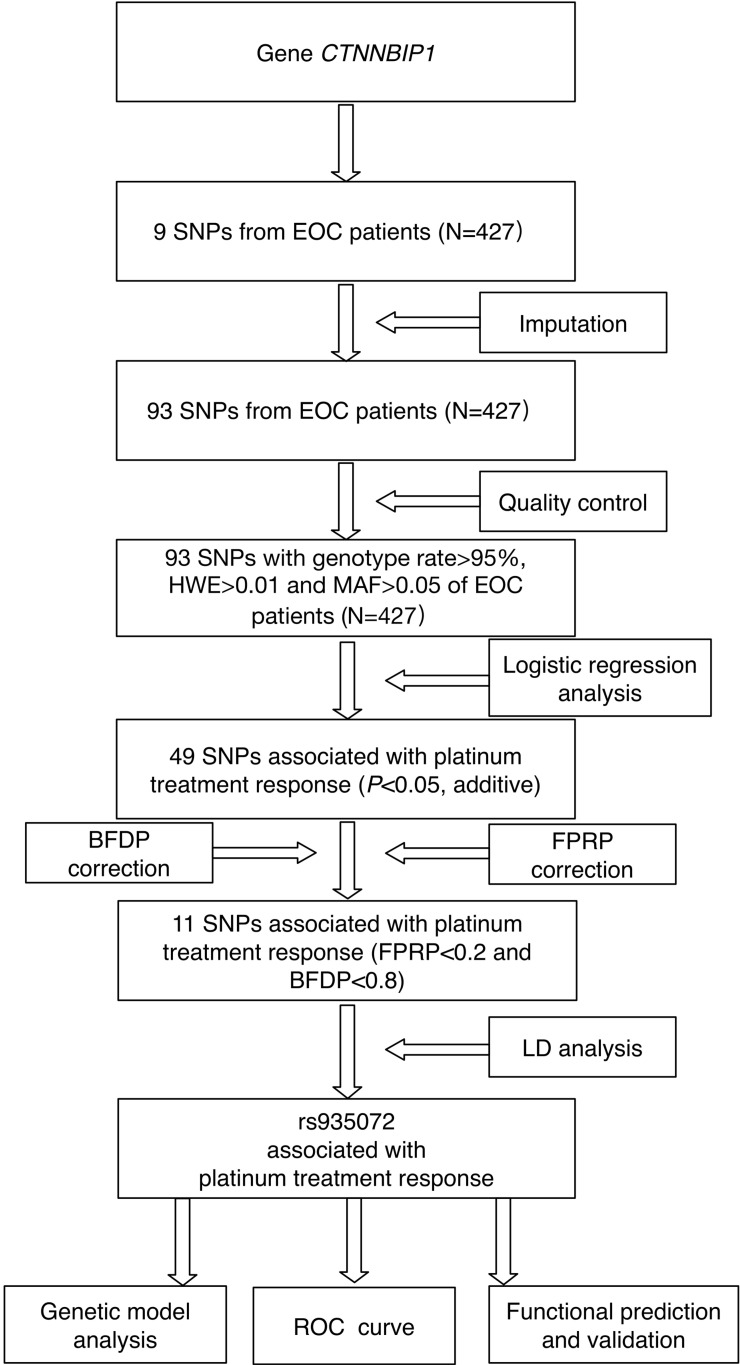
The study analysis flowchart.

The study flowchart is shown in **Figure 1**. The *CTNNBIP1* genotyping data (nine SNPs) of 427 patients who underwent platinum-based treatment were available for subsequent analysis. After quality control and imputation, 93 SNPs in *CTNNBIP1* were included for further analysis, of which nine SNPs were genotyped and 84 were imputed. As a result, we found that 49 SNPs in *CTNNBIP1* were individually and significantly associated with the response to platinum treatment of EOC patients (adjusted *P*<0.05). To control for the probability of false positive associations with platinum treatment response of EOC patients, both FPRP and BFDP were performed and 11 SNPs passed the corrections (**Table S1**), although false discovery rate (FDR) was not applied because of the high LD among the SNPs as a result of imputation. The following LD analysis revealed that these 11 SNPs were in LD (r^2^>0.8), one of which, *CTNNBIP1* rs935072A>T, is located at the 3'- UTR of the gene and was selected for further genetic modeling and functional validation. *CTNNBIP1* rs935072A>T was found to be associated with platinum treatment response ([adjusted odds ratio (OR)] = 0.62, 95% confidence interval (CI) = 0.44-0.88, *P=*0.009 and FPRP=0.164, BFDP=0.568). Furthermore, we performed the stepwise multivariate logistic regression analysis to select the optimal predictors of platinum treatment response in EOC patients, with adjustment for other clinical variables including age, FIGO stage, histology, grade, residue (optimal debulking <1 cm), ascites and neoadjuvant chemotherapy. The above-mentioned *CTNNBIP1* rs935072 SNP still remained noteworthy (**Table S2**). In addition to age (adjusted OR=1.08, 95% CI=0.99-1.18, *P*=0.057), FIGO stage (adjusted OR=1.15, 95% CI=1.03-1.29, *P*=0.015), residue (adjusted OR=1.31, 95% CI=1.15-1.49, *P*<0.001), the *CTNNBIP1* rs935072A>T remained an independent predictive factor (adjusted OR=0.92, 95% CI=0.87-0.98, *P*=0.014) of platinum treatment response in EOC patients.

### Genetic association with platinum treatment response of EOC patients

The genotype (A/A, A/T, and T/T) frequencies of the* CTNNBIP1* rs935072 SNP were 50.6%, 39.6%, and 9.8%, respectively, and the allele A and T frequencies were 70.4% and 29.6%, respectively. In the multivariate analysis with adjustment for age, FIGO stage, histology, grade, residue (optimal debulking <1 cm), ascites and neoadjuvant chemotherapy, the *CTNNBIP1* rs935072 AT/TT genotypes were associated with a decreased risk of developing chemoresistance (adjusted OR=0.89, 95% CI=0.82-0.97 and *P*=0.010), compared with the AA genotype (**Table 1**). Then, we used diagnostic ROC curve to assess discriminative accuracy of the identified SNP *CTNNBIP1* rs935072A>T. First, we constructed a logistic regression model with the above-mentioned clinical variables. Then, we added rs935072 to the model in an additive genetic model. Although the ROC prediction model incorporating *CTNNBIP1* rs935072 was borderline significantly different from that incorporating only clinical factors, the AUC was increased (all patients: AUC 0.671 vs. 0.692, 95% CI=0.615-0.726 vs. 0.638-0.747, *P*=0.057, **Figure S2**).

### Stratified analysis between unfavorable genotypes and platinum treatment response

Then, we performed stratified analysis to select the subgroup of patients who could benefit more from the identified SNP in the combination with other predictors (**Table 2**), including age, tumor grade, histology, FIGO stage, residue, ascites as well as neoadjuvant chemotherapy. We found that *CTNNBIP1* rs935072A>T was more effective to predict the platinum response in subgroups of patients who were older than 54 (adjusted OR=0.83, 95% CI=0.73-0.95, *P*=0.006), with a high grade (adjusted OR=0.90, 95% CI=0.83-0.99, *P*=0.022) or serous tumors (adjusted OR=0.83, 95% CI=0.72-0.97, *P*=0.020), with an advanced tumor stage (adjusted OR=0.88, 95% CI=0.80-0.98, *P*=0.017), and without neoadjuvant chemotherapy (adjusted OR=0.87, 95% CI=0.80-0.96, *P*=0.005). No interactive effects between covariates and platinum treatment response-associated genotypes were identified, except for an interaction between *CTNNBIP1* rs935072 and tumor grade (*P*=0.037).

### Functional prediction of *CTNNBIP1* rs935072A>T

**Figure 2:**
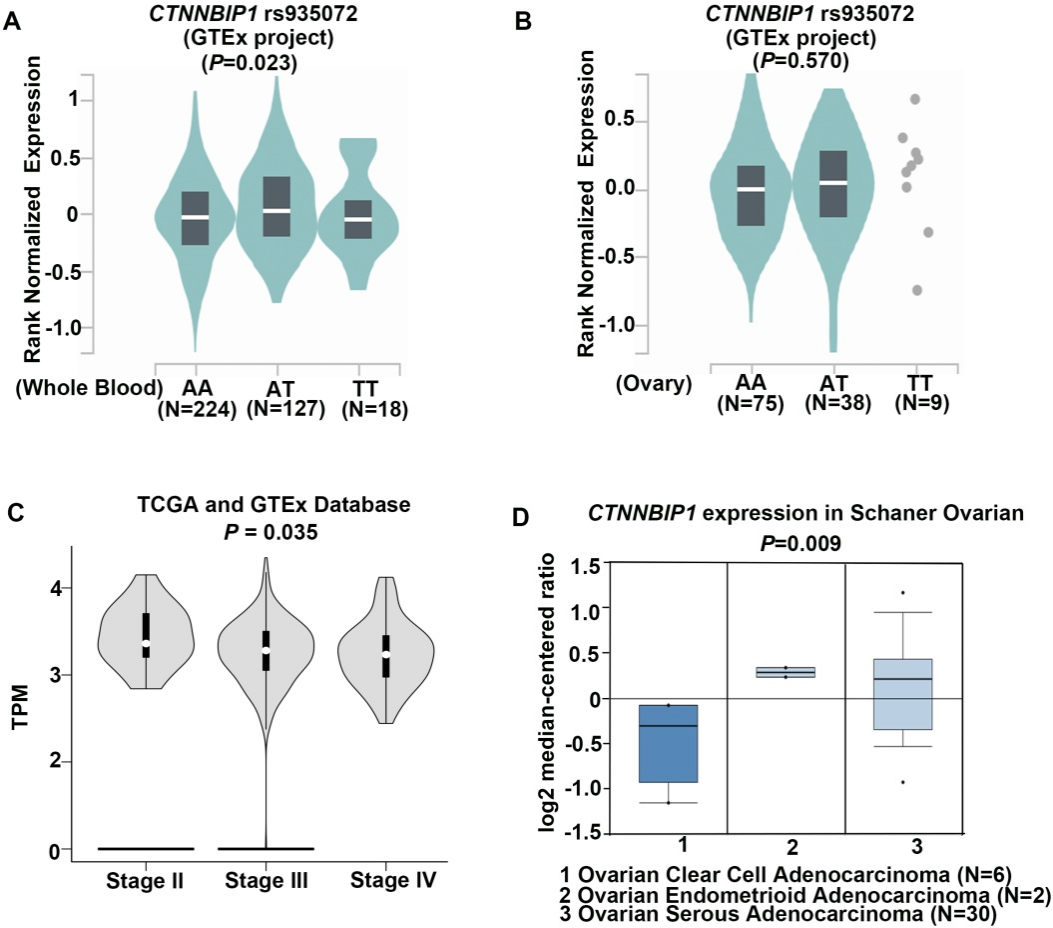
** Functional prediction of genetic variants of *CTNNBIP1.* A,** The impact of *CTNNBIP1* rs935072 SNP on the mRNA expression of *CTNNBIP1* in whole blood tissues from the GTEx database. **B,** The impact of *CTNNBIP1* rs935072 SNP on the mRNA expression of *CTNNBIP1* in ovary tissues from the GTEx database. c, *CTNNBIP1* expression in different stage of ovarian cancer tissues from TCGA database. TPM, Transcripts per Kilobase of an exon model per Million mapped reads. **C,** *CTNNBIP1* expression in different histology of ovarian cancer tissues from Oncomine database.

To investigate the role of *CTNNBIP1* rs935072A>T in regulating its gene expression, we searched the GTEx database where both mRNA expression and genotype data were available. Initial analysis found that the mRNA expression levels were up-regulated by the *CTNNBIP1* rs935072 A>T change in whole blood cells (*P***=**0.023, **Figure 2A**). Despite no statistical change in ovary tissues, patients (n=38) with the AT genotype showed an increased *CTNNBIP1* mRNA expression level compared with those (n=75) with the AA genotype (*P*=0.570, **Figure 2B**). We also performed the expression quantitative trait loci analysis for the correlation between the SNP and mRNA expression levels by using data in the 1000 Genomes Project (n=373 and *P*=0.937, **Figure S3A**) and the Hapmap3 project of Chinese subjects (n=79 and *P*=0.671, **Figure S3B**). Unfortunately, no significant difference in the expression levels was observed by the A>T change due to the relative small number of patients.

We further searched in the TCGA database for the mRNA expression levels of *CTNNBIP1* in both EOC and normal ovarian tissues, but there was no difference in *CTNNBIP1* mRNA expression levels between EOC and normal ovarian tissues. However, we found that *CTNNBIP1* mRNA expression was down-regulated as the stage increased (**Figure 2C**), indicating its role in tumor progression. In addition, because ovarian serous carcinoma is the most sensitive type to common platinum-based chemotherapy than other types such as mucinous 29 or clear cell carcinoma 30, we then searched the Oncomine database to analyze mRNA expression levels of *CTNNBIP1* by different histology subtypes of ovarian cancer. Strikingly, the mRNA expression levels of *CTNNBIP1* was much higher (*P*=0.009) in serous tumor type (n=30) than in other types (n=8) (**Figure 2D**). Taken together, the underlying protective mechanism of the rs935072 A>T may be attributed to altered mRNA expression levels of *CTNNBIP1*.

### The effect of *CTNNBIP1* rs935072 A>T on the binding ability of miR-27a-3p and 3'- UTR of *CTNNBIP1*

**Figure 3:**
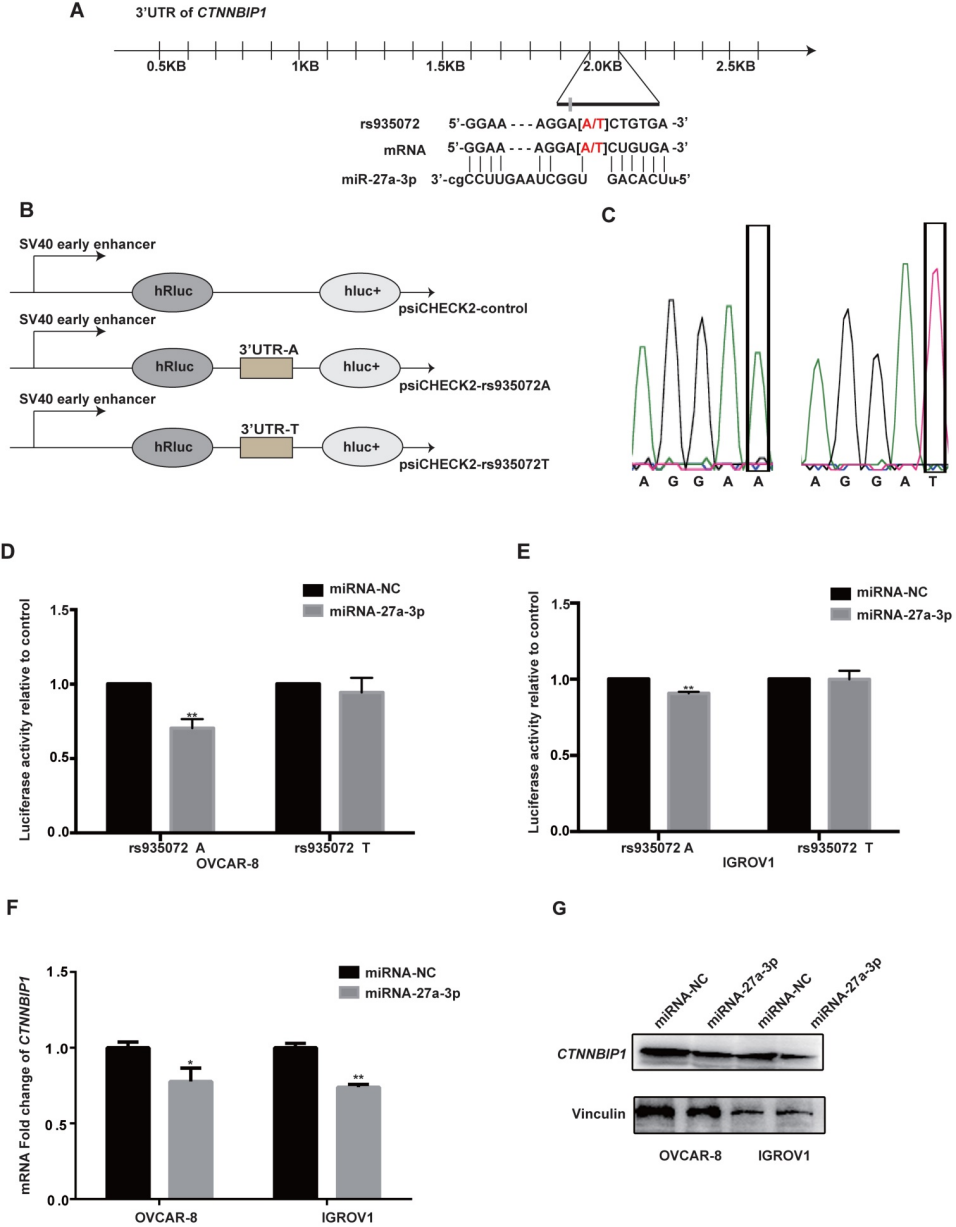
** The *CTNNBIP1* rs935072 A>T contributes to the decreased binding affinity of miR-27a-3p to the *CTNNBIP1* 3'UTR and increased the expression of *CTNNBIP1.* A,** Graphic representation of the detailed location of rs935072 in the 3'UTR of *CTNNBIP1*, which is also at the miRNA-binding site with the A allele. **B,** Schematic drawing of the luciferase reporter system. **C,** Sequencing results of the Psi-CHECK2 vector containing rs935072 A or T allele. Abbreviation: Mut, Mutagenesis. **D-E,** Luciferase activity in the presence of the miR-27a-3p transfected into IGROV1 and OVACR-8 cell lines. F, The expression of *CTNNBIP1* was detected by the qRT-PCR assay in IGROV1 and OVACR-8 cells overexpressing miR-27a-3p and control cells. **G,** The expression of *CTNNBIP1* was detected by western blot assay in IGROV1 and OVACR-8 cells overexpressing miR-27a-3p and control cells. * *P* < 0.05. ** *P* < 0.01.

Then, we explored putative function of *CTNNBIP1* rs935072 located at the 3'UTR, which is a critical binding site of microRNA. We hypothesized that the rs935072 A>T change might reduce the binding capacity of miRNA, thereby affecting the expression of *CTNNBIP1* and platinum treatment response of EOC patients. Therefore, we used the online tools of MirSNP (http://bioinfo.bjmu.edu.cn/mirsnp/search/), TargetScan (http://www.targetscan.org/vert_71/) and SNPInfo (https://snpinfo.niehs.nih.gov/) to predict the specific microRNA binding with the *CTNNBIP1* 3'UTR. The results showed that miR-27a-3p was a potential microRNA that could bind to the 3'UTR of *CTNNBIP1* **(Figure 3A)**. Specifically, the *CTNNBIP1* rs935072 A>T change may affect the binding of *CTNNBIP1* 3'UTR with miR-27a-3p. To test this hypothesis experimentally, we constructed a Psi-CHECK2 vector carrying the 3'UTR of *CTNNBIP1* with rs935072 A or T allele (**Figure 3B**). The sequencing results of the Psi-CHECK2 vector containing either rs935072 A or T allele are shown in **Figure 3C**. In both IGROV1 and OVCAR-8 cell lines, when overexpressing miR-27a-3p, we observed a down-regulated luciferase activity associated with the rs935072 A allele in the luciferase reporter assay, and this effect was abrogated when the rs935072 A allele changed into the T allele (**Figure 3D and 3E**). In addition, we found that the miR-27a-3p mimic could significantly reduce both mRNA and protein expression levels of *CTNNBIP1* (**Figure 3F-3G**). Together with the results that rs935072 A>T change affected the efficiency of microRNA-27a-3p binding, we speculated that a weak binding affinity of miR-27a-3p to the 3'UTR region may result in the up-regulation of the *CTNNBIP1* effectively.

### CTNNBIP1 sensitizes ovarian cancer cells to cisplatin

**Figure 4:**
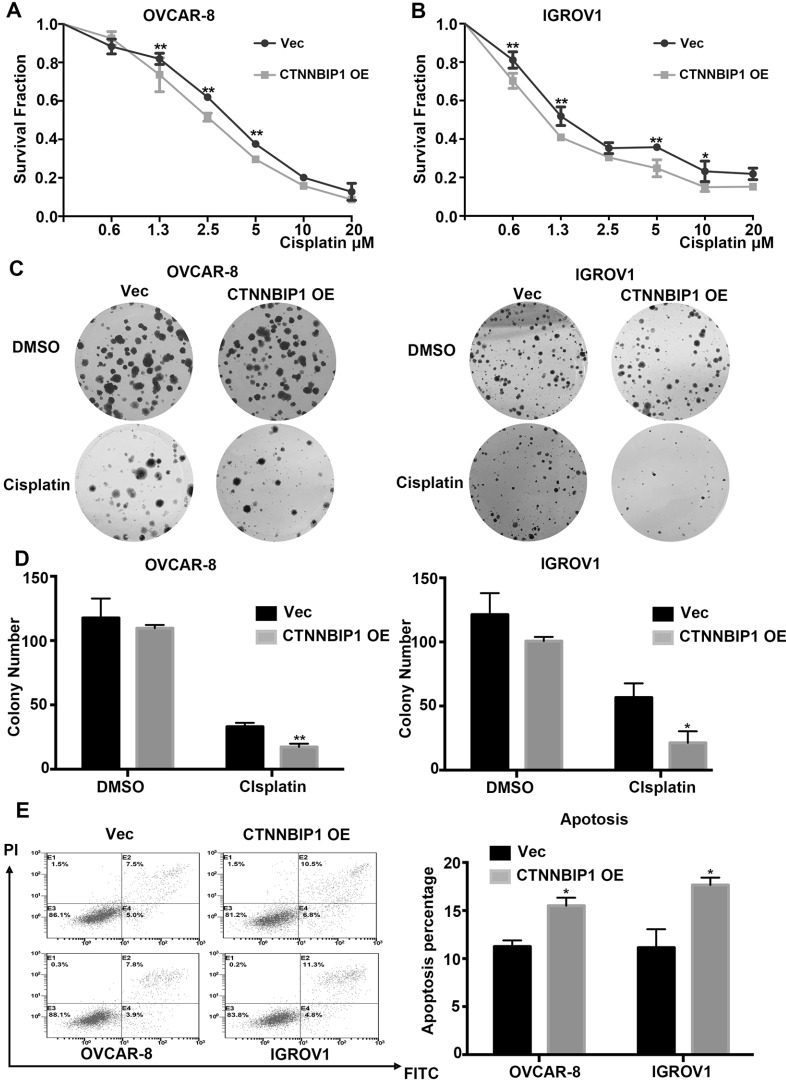
***CTNNBIP1* sensitizes ovarian cancer cells to cisplatin. A-B,** Cell Counting Kit-8 assays showed the effect of empty vector and *CTNNBIP1* overexpression on the chemosensitivity of ovarian cancer cells to the cytotoxic effect of cisplatin. **C-D,** Colony formation assays showed the effect of empty vector and *CTNNBIP1* overexpression on the chemosensitivity of ovarian cancer cells to the cytotoxic effect of cisplatin. **E,** The percentage of apoptotic cells in indicated group after cisplatin treatment. Cells were stained with annexin V-fluorescein isothiocyanate (FITC) and propidium iodide (PI) to detect cells in early apoptosis (annexin V+ PI-) and late apoptosis (annexin V+ PI+). Representative pictures are shown. * *P* < 0.05. ** *P* < 0.01.

To further explore the effect of CTNNBIP1 on chemotherapy sensitivity of ovarian cancer, we treated IGROV1 and OVCAR-8 cell lines selectively overexpressing CTNNBIP1 and their corresponding control cells with different concentrations of cisplatin for 48 h. The Cell Counting Kit-8 assays (**Figure 4A-4B**) revealed that the overexpression of CTNNBIP1 increased the sensitivity to cisplatin. Colony formation assay results clearly showed that the overexpression of CTNNBIP1 reduced the number and size of the colonies after treatment with cisplatin (**Figure 4C-4D**). Then, we performed flow cytometry analysis to measure apoptotic cells 48 hours after treatment with cisplatin. Compared with control cells, the overexpression of CTNNBIP1 induced more cell death (**Figure 4E**) in all cell lines tested. Taken together, our experimental data suggested that CTNNBIP1-overexpressing cells had a higher rate of apoptosis than control cells in response to cisplatin treatment and that CTNNBIP1 over-expression synergized with cisplatin to inhibit cell proliferation.

## Discussion

In the present study, we found that 11 out of the single-locus analysis of 93 *CTNNBIP1* SNPs were associated with chemotherapy response. In the LD analysis, the *CTNNBIP1* rs935072 A>T SNP captured other 10 SNPs in a high LD block. Further stepwise multivariate logistic regression analysis showed that the *CTNNBIP1* rs935072 A>T SNP was an independent predictor for treatment response of EOC patients. Results of the genotype-phenotype correlation analysis further demonstrated that the rs935072 T allele was associated with increased mRNA expression levels of *CTNNBIP1*, a potential molecular marker for EOC patients to overcome chemoresistance.

The Wnt/β-catenin signaling is an evolutionarily conserved and versatile pathway that is associated with many cellular processes and a wide variety of human diseases, especially for cancer 31. β-Catenin serves as a core component in the Wnt/β-catenin signaling. The stabilization of β-catenin is critical in cancer stem cell survival 32 and chemoresistance 33. The β-catenin tends to accumulate in the cytoplasm and then is translocated into the nucleus, when aberrant *WNT* activation and mutations of the *WNT* gene or destruction of the complex components occurred 34. Consequently, β-catenin binds to TCF/LEF and some other co-regulators to promote the transcription of target genes such as *c-Myc* 35 and *cyclin D1* 36, which initiate tumorigenesis. There are many negative regulation mechanisms of β-catenin. CTNNBIP1 is one of the molecules that inhibit β-catenin from forming a complex with TCF/LEF and then inactivates the transcription of Wnt target genes, thereby negatively regulating the Wnt/β-catenin pathway 15. Here, we reported a novel regulatory mechanism of *CTNNBIP1* activation, thereby uncovering a potential chemotherapeutic target for ovarian cancer treatment.

To date, few studies have reported the role of genetic variants in regulating the expression of *CTNNBIP1*, although rare somatic mutations in *CTNNBIP1* were observed in some studies of malignancies 37, 38. For example, in breast cancer, overall alterations (52-55%) with frequent methylation (44-45%) and deletion (20-32%) of *CTNNBIP1* were observed 39. However, no studies explored the role of *CTNNBIP1* SNPs in treatment response in ovarian cancer patients. To the best of our knowledge, the present study is the first that investigated associations between SNPs of *CTNNBIP1* and the response to platinum treatment of Chinese EOC patients with functional validation, which provides an additional evidence to support the role of *CTNNBIP1* rs935072 in predicting platinum-treatment response of Chinese EOC patients.

SNPs located at the gene 3'UTRs possibly affect cancer development and progression via regulating the efficiency of miRNA binding to the specific sites 40, 41. Previous studies demonstrated that miR-214 42 and miR-603 43 negatively regulated the expression levels of *CTNNBIP1.* In the present study, by using *in silico* tools, we identified miR-27a-3p as a new potential regulator of *CTNNBIP1* expression*.* Since *CTNNBIP1* rs935072 appears to be at a miR-27a-3p binding site, we observed a down-regulated luciferase activity for the rs935072 A allele, compared with that for the T allele in both IGROV1 and OVCAR-8 cell lines. The observed decrease in luciferase activities meant an increased binding capacity of miR-27a-3p, thereby affecting the expression of *CTNNBIP1*. Furthermore, from the TCGA and Oncomine databases, we found that *CTNNBIP1* might act as a chemosensitivity-related gene due to its relatively higher expression levels in chemosensitive ovarian cancer serious histology, compared with other types of histology. Further functional experiments confirmed that the overexpression of *CTNNBIP1* sensitized ovarian cancer cell to cisplatin treatment. These results showed that the *CTNNBIP1* rs935072 T allele may account for a good platinum response of EOC patients by modulating its mRNA expression.

Our findings may help identify subgroups of patients at a higher risk of developing chemoresistance and those patients who are more likely to benefit from individualized treatment strategies. However, the present study had several limitations. First of all, the inherent limitation was the retrospective study design with inevitable selection bias. Secondly, the sample size of study population was relatively small, which had limited statistical power to detect the true weak effect of SNPs on chemotherapeutic response in the analysis. Moreover, patients enrolled in the present study were from single cancer center and thus could not represent the general EOC patient population in China. Future multi-center validation is needed to substantiate our findings. Finally, additional *in vivo* studies are required to confirm our observed mechanism of *CTNNBIP1* in platinum treatment response of ovarian cancer.

In conclusion, in the present study, we identified *CTNNBIP1* rs935072 to be a potential biomarker for predicting platinum chemotherapeutic response for EOC patients. The underlying mechanism for the effect of the *CTNNBIP1* rs935072 A>T change on chemotherapy treatment response resulted from a lower binding affinity of miR-27a-3p, thereby leading to the up-regulation of the* CTNNBIP1* expression. Further *in vitro* experiments demonstrated that overexpression of CTNNBIP1 sensitized ovarian cancer cells to platinum treatment. Once further validated by other investigators, these findings would provide novel clues for individualized therapy for EOC patients in the future.

## Supplementary Material

Supplementary figures and tables.

## Figures and Tables

**Table 1:** Association of *CTNNBIP1* rs935072 with platinum treatment response of EOC patients

Genetic variant	Genotype	Number/Event	Platinum treatment response			
			Univariate		Multivariate	
			OR (95%CI)	*P*	OR (95%CI)	P*
*CTNNBIP1*	AA	216/80	1.00		1.00	
rs935072 A>T	AT	169/45	0.90 (0.82-0.99)	**0.029**	0.90 (0.82-0.98)	**0.022**
	TT	42/9	0.86 (0.73-0.99)	**0.046**	0.87 (0.75-1.02)	0.079
Dominant model	AA/AT +TT	211/54	0.89 (0.82-0.97)	**0.011**	0.89 (0.82-0.97)	**0.010**
Recessive model	AA+AT/TT	385/125	0.90 (0.77-1.04)	0.144	0.92 (0.79-1.06)	0.234
Additive model	AA/AT/TT		0.92 (0.86-0.98)	**0.010**	0.92 (0.86-0.98)	**0.013**

Abbreviations: EOC, epithelial ovarian carcinoma; OR, odds ratio; CI, confidence interval; P*, the multivariate logistic regression analyses were adjusted for age, tumor grade, histological types, FIGO stage, residue, ascites and neoadjuvant chemotherapy; The results were in **bold** if *P*<0.05.

**Table 2:** Stratification analysis for associations between *CTNNBIP1* rs935072 A>T and platinum treatment response of the EOC patients

Variables	*CTNNBIP1* rs935072 (Number/Event)		Platinum treatment response		
	AA	AT & TT	OR (95% CI)	P^*^	*P*^†^
**Age at diagnosis**					0.176
<54	116/34	112/27	0.94 (0.84-1.06)	0.326	
≥54	100/46	99/27	0.83 (0.73-0.95)	**0.006**	
**Grade**					**0.037**
Low	6/3	7/0	0.43 (0.22-0.84)	0.089	
High	209/77	204/54	0.90 (0.83-0.99)	**0.022**	
**Histological types**					0.278
Serous	151/55	143/40	0.92 (0.83-1.02)	0.105	
Others‡	65/25	68/14	0.83 (0.72-0.97)	**0.020**	
**FIGO Stage**					0.620
I - II	39/7	35/5	0.93 (0.77-1.12)	0.435	
III - IV	170/71	158/47	0.88 (0.80-0.98)	**0.017**	
**Residue disease**					1.000
≤1 cm	143/42	133/28	0.92 (0.83-1.02)	0.105	
>1 cm	28/17	27/14	0.92 (0.83-1.02)	0.105	
**Ascites**					0.451
No	33/9	26/2	0.85 (0.70-1.04)	0.129	
Yes	130/50	130/40	0.93 (0.83-1.04)	0.202	
**Neoadjuvant**					0.291
No	175/67	177/43	0.87 (0.80-0.96)	**0.005**	
Yes	41/13	34/11	0.99 (0.80-1.23)	0.922	

Abbreviations: EOC, epithelial ovarian carcinoma; OR, odds ratio; CI, confidence interval; P^*^ - *P* value of multivariate logistic regression analyses was adjusted for age, tumor grade, histological types, FIGO stage, residue, ascites and neoadjuvant chemotherapy; *P*^†^ -* P* value of Cochran's Q test for heterogeneity between the two groups; ^‡^ - other histological types include mucinous, endometrioid, clear cell and others types of EOC; The results were in **bold** if *P*<0.05 (the stratified factor in each stratum excluded).

## Abbreviations

EOC: epithelial ovarian cancer
SNPs: single nucleotide polymorphisms
OR: odds ratio
CI: confidence interval
GWASs: genome-wide association studies
*CTNNBIP1*: Catenin beta interacting protein 1
TCF/LEF: T-cell factor/lymphoid enhancer factor
FUSCC: Fudan University Shanghai Cancer Center
SOCS: Shanghai Ovarian Cancer Study
FIGO: International Federation of Gynecology and Obstetrics
FDR: False Discovery rate
FPRP: false-positive report probability
BFDP: Bayesian false-discovery probability
LD: linkage disequilibrium
ROC: Receiver operating characteristic
AUC: area under the curve
DMEM: Dulbecco's modified Eagle's medium
SD: standard deviation
