# Supplementary material for: Functional genetic variants of CTNNBIP1 predict platinum treatment response of Chinese epithelial ovarian cancer patients
Source: J Cancer. 2020 Sep 30;11(23):6850–60. doi: 10.7150/jca.48218 (PMC7592014; doi:10.7150/jca.48218)

Table S1. SNPs of *CTNNBIP1* associated with platinum treatment response of EOC patients

					Platinum treatment response				
**SNP**	**position**	**A1**	**A2**	**MAF**	**OR (95%CI)**	P^*	**FDR**	**FPRP**	**BFDP**
rs1220405	9922245	A	G	0.305	0.61 (0.43-0.86)	**0.005**	1.00	0.124	0.446
rs935073	9923741	A	G	0.306	0.62 (0.44-0.88)	**0.007**	1.00	0.164	0.535
rs946275	9924957	C	A	0.305	0.62 (0.44-0.88)	**0.007**	1.00	0.164	0.535
rs1220418	9938819	C	T	0.305	0.62 (0.44-0.88)	**0.007**	1.00	0.164	0.535
rs935075	9942118	C	T	0.305	0.62 (0.44-0.88)	**0.007**	1.00	0.164	0.535
rs1220381	9948405	T	C	0.305	0.62 (0.44-0.88)	**0.007**	1.00	0.164	0.535
rs3120822	9955216	C	T	0.305	0.62 (0.44-0.88)	**0.007**	1.00	0.164	0.535
rs1220384	9956185	C	A	0.305	0.62 (0.44-0.88)	**0.007**	1.00	0.164	0.535
rs1220412	9926229	T	C	0.324	0.63 (0.45-0.88)	**0.007**	1.00	0.141	0.518
rs935072	9910357	A	T	0.298	0.63 (0.44-0.89)	**0.009**	1.00	0.174	0.568
rs2486084	9965425	G	A	0.323	0.63 (0.45-0.89)	**0.008**	1.00	0.174	0.568

Abbreviations: SNPs, single nucleotide polymorphisms; A1, Allele 1, the major allele; A2, Allele 2, the minor allele; EOC, epithelial ovarian carcinoma; OR, odds ratio; CI, confidence interval; FDR, false discovery rate; FPRP, false-positive report probability; BFDP, Bayesian false-discovery probability; MAF, minor allele frequency;

P^*, obtained in multivariate logistic regression analysis with variables including age, tumor grade, histological types, FIGO stage, residue, ascites and neoadjuvant chemotherapy.

*T*he results were in **bold,** if *P*<0.05

Table S2. Stepwise multivariate logistic regression analysis for the identification of significant SNPs in EOC patients

**Variables**	**Category**	**Frequency**	**OR (95%CI)**	***P****
Age at diagnosis	≤54 / >54	228/199	1.08 (0.99-1.18)	0.057
FIGO Stage	I-II / III-IV	74/328	1.15 (1.03-1.29)	**0.015**
Residue	≤1cm / >1cm	276/55	1.31 (1.15-1.49)	**<0.001**
rs935072	AA / AT / TT	216/169/42	0.92 (0.87-0.98)	**0.014**

Abbreviations: SNPs, single nucleotide polymorphisms; EOC, epithelial ovarian carcinoma; OR, odds ratio; CI, confidence interval; FIGO, international federation of gynecology and obstetrics;

P^*, obtained in a stepwise multivariate logistic regression analysis, and variables included age, tumor grade, histological types, FIGO stage, residue, ascites, neoadjuvant chemotherapy and *CTNNBIP1* rs935072;

The results were in bold, if $P<0.05$.

Supplementary Figure 1

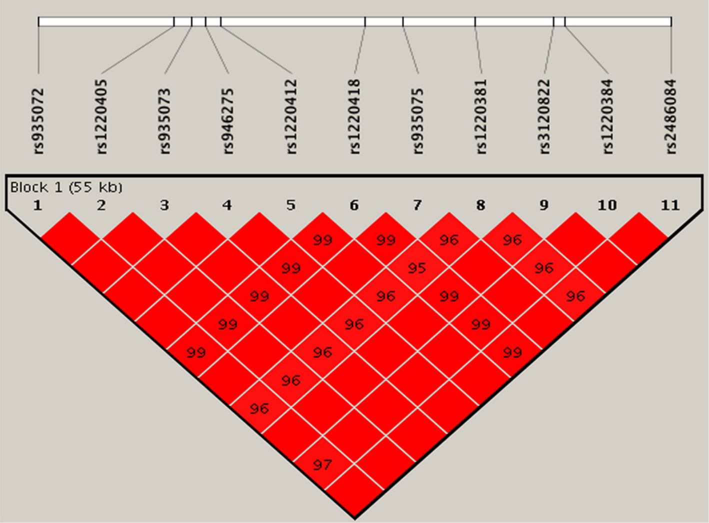

Supplementary Figure 2

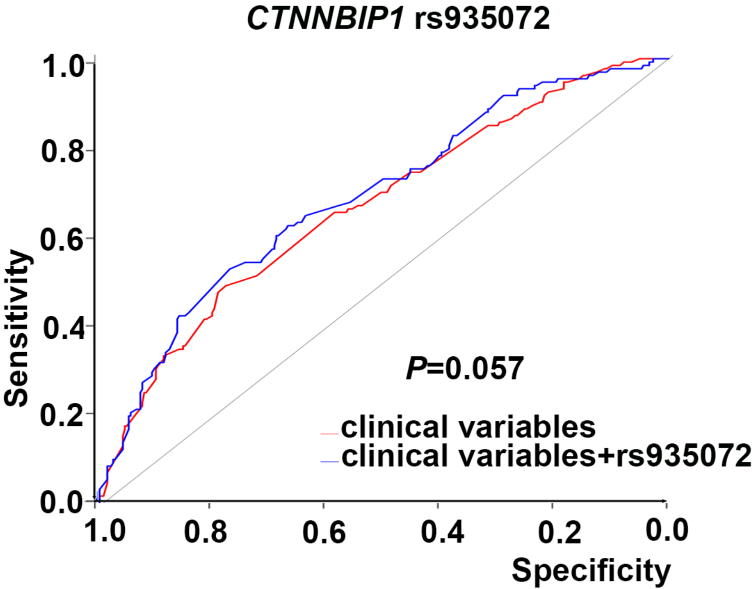

Supplementary Figure 3

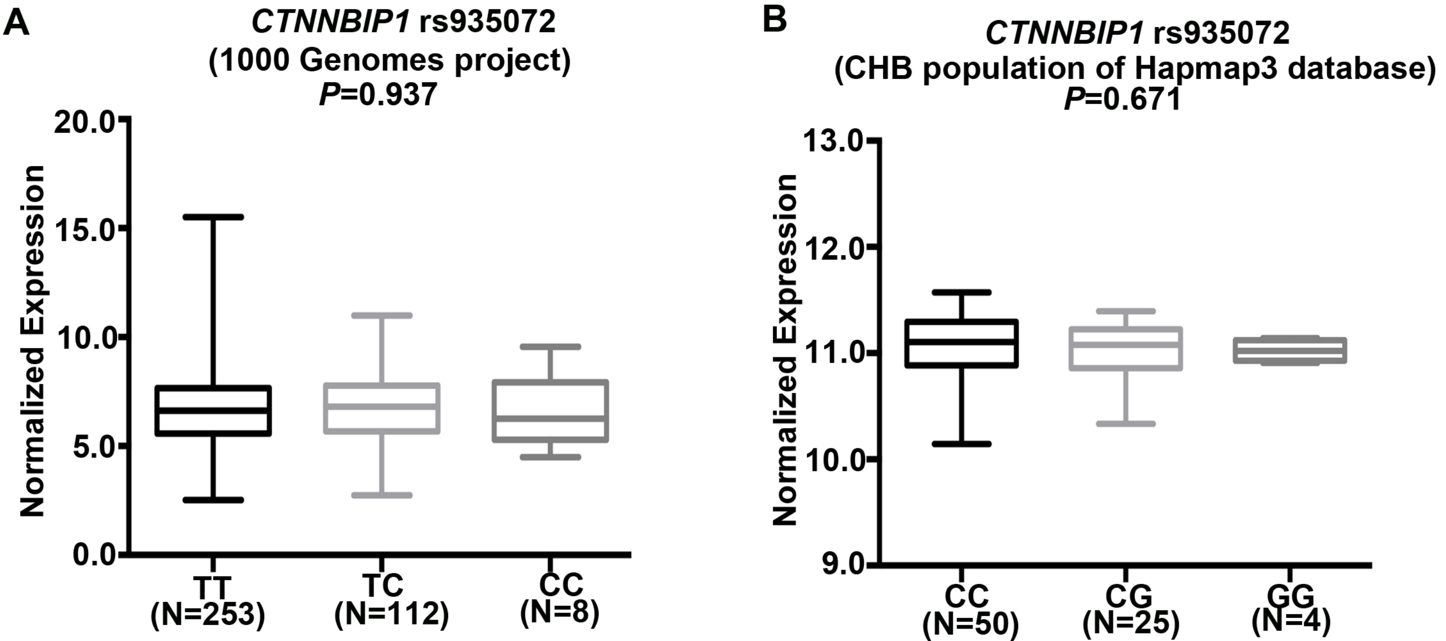

Supplement: Supplementary file 1 — Supplementary figures and tables. [file jcav11p6850s1.pdf]
